# Patient-Level Mass Findings on Mammography and Ultrasonography in Ductal Carcinoma In Situ: Association with Invasive Carcinoma

**DOI:** 10.3390/diagnostics16132062

**Published:** 2026-07-01

**Authors:** Ozlem Unal, Servet Kocaoz, Gulay Gencer, Eda Sener Alcın, Berkan Alcın, Fazlı Erdogan

**Affiliations:** 1 Radiology, Ankara Yıldırım Beyazıt University, Ankara 06800, Turkey; 2 General Surgery, Ankara Bilkent City Hospital, Ankara 06800, Turkey; servet.kocaoz@gmail.com; 3 Radiology, Ankara Bilkent City Hospital, Ankara 06800, Turkey; gulaysulugencer@gmail.com (G.G.); dredasener@gmail.com (E.S.A.); berkanalcin@gmail.com (B.A.); 4 Pathology, Ankara Yıldırım Beyazıt University, Ankara 06760, Turkey; fazlierdogan@gmail.com

**Keywords:** ductal carcinoma in situ, invasive carcinoma, mammography, ultrasonography, multimodal imaging, mass lesion, breast imaging

## Abstract

**Background:** Preoperative identification of invasive carcinoma in patients with ductal carcinoma in situ (DCIS) remains challenging. While individual imaging findings have been widely investigated, the significance of mass findings identified on both mammography and ultrasonography remains less clearly defined. **Methods:** This retrospective study included 125 patients with DCIS who underwent both mammography and ultrasonography (US). Imaging findings were categorized as no mass on either modality, mass detected on a single modality, or mass findings identified on both mammography and ultrasonography. Associations between imaging patterns and invasive carcinoma were evaluated using descriptive analyses. **Results:** The proportion of invasive carcinoma was highest among patients with mass findings identified on both mammography and ultrasonography. Invasive carcinoma was observed in 6 of 71 patients (8.5%) with no mass on either modality, 5 of 37 patients (13.5%) with a mass detected on a single modality, and 8 of 17 patients (47.1%) with mass findings identified on both mammography and ultrasonography. However, substantial overlap between imaging categories remained present. **Conclusions:** Mass findings identified on both mammography and ultrasonography were more frequently observed in DCIS cases with invasive carcinoma. However, substantial overlap between imaging categories remained present, limiting reliable distinction at the individual patient level.

## 1. Introduction

Ductal carcinoma in situ (DCIS) is a non-invasive breast neoplasm characterized by the proliferation of malignant epithelial cells confined to the ductal system. With the widespread use of screening mammography, its detection has increased substantially, and DCIS now represents a considerable proportion of newly diagnosed breast malignancies [1,2].

Although DCIS is considered a non-invasive entity, invasive carcinoma is identified on final surgical pathology in a subset of cases initially diagnosed as DCIS, with reported underestimation rates ranging from approximately 10% to 30% [3,4]. This underestimation has been recognized as a challenge in the management of selected patients with DCIS [5,6]. In addition, DCIS demonstrates heterogeneous clinical behaviour, with variable risks of progression and long-term outcomes [7,8].

Identifying invasive disease before surgery remains difficult in many patients with DCIS. Various clinicopathological factors have been investigated, but findings have not been consistent, and management strategies continue to vary across clinical settings [7,9,10]. Imaging plays an important role in this assessment. Mammography most commonly demonstrates microcalcifications, although mass lesions, asymmetry, and architectural distortion may also be observed [11]. Ultrasonography (US) provides complementary information, particularly in the evaluation of mass lesions, but its findings are often non-specific when interpreted alone [12]. Previous studies have reported that mass findings are more frequently observed in DCIS cases with invasive carcinoma [11,13]. However, most previous studies have evaluated mammographic and ultrasonographic findings separately, and the relationship between mass findings identified on both imaging modalities and invasive carcinoma has not been fully clarified.

The aim of the present study was to evaluate whether mass findings identified on both mammography and ultrasonography are more frequently observed in patients with DCIS associated with invasive carcinoma [10].

## 2. Materials and Methods

### 2.1. Study Design and Patients

This retrospective single-center study included patients diagnosed with ductal carcinoma in situ (DCIS) who underwent surgical treatment between March 2019 and March 2026. Only patients with histopathologically confirmed DCIS and available preoperative mammography and ultrasonography (US) were included. Cases with missing clinical, radiological, or pathological data were excluded.

A total of 152 patients were initially identified. After excluding patients with incomplete imaging or histopathological data (*n* = 11) and those whose imaging studies could not be retrieved (*n* = 16), 125 patients were included in the final analysis.

**Figure 1:**
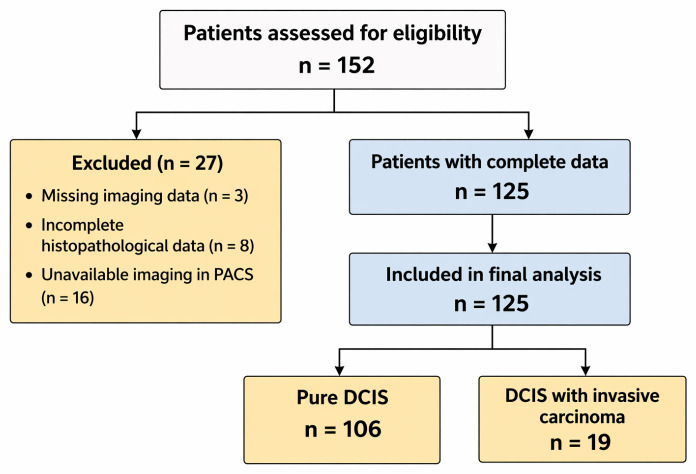
Retrospective patient selection flow diagram. A total of 152 patients with DCIS were initially identified. After exclusions due to incomplete imaging, unavailable imaging retrieval, or missing histopathological data, 125 patients were included in the final analysis.

Based on final surgical pathology, patients were classified as having either pure DCIS or DCIS with an invasive component. The patient selection process is summarized in Figure 1.

### 2.2. Radiological Evaluation

Preoperative mammographic and ultrasonographic examinations were reviewed retrospectively by a breast radiologist with more than 15 years of experience who was blinded to the final histopathological results at the time of image evaluation.

Mammographic findings were assessed according to the BI-RADS lexicon, including microcalcifications, masses, asymmetry, and architectural distortion. Breast density was recorded using BI-RADS categories (A–D). Ultrasonographic findings were evaluated using standard BI-RADS terminology, including solid masses, complex masses, and ductal abnormalities.

For the purposes of this study, the analysis focused on the presence of a mass on both mammography and ultrasonography. The presence of mass findings identified on both mammography and ultrasonography was defined as the identification of at least one mass finding on each modality at the patient level. Formal lesion-by-lesion matching was not performed because of the retrospective design and variability in imaging documentation across modalities. Therefore, this imaging category was evaluated at the patient level rather than through direct lesion-by-lesion correlation. The analysis was intentionally limited to the presence of a mass, without incorporating more detailed BI-RADS descriptors such as margin or shape.

Imaging findings were grouped into three categories: no mass on either modality, mass detected on a single modality, and mass findings identified on both mammography and ultrasonography. This classification was intended to reflect patient-level imaging patterns rather than individual lesion characteristics.

All imaging evaluations were performed by a single reader. While this ensured consistency, interobserver variability was not assessed and should be considered when interpreting the results.

### 2.3. Sentinel Lymph Node Biopsy

Sentinel lymph node biopsy (SLNB) was routinely performed in patients with confirmed invasive carcinoma. In patients with a preoperative diagnosis of DCIS, SLNB was performed selectively based on clinical and imaging findings. In patients with a preoperative diagnosis of DCIS, SLNB was generally considered in the presence of imaging findings suspicious for invasion, planned mastectomy, large lesion extent, or clinical concern raised during multidisciplinary evaluation. This selective approach may have influenced the observed rates of nodal involvement.

On ultrasonography, features such as cortical thickening and hilar irregularity were considered suggestive of nodal involvement.

### 2.4. Histopathological Evaluation

Final histopathological diagnoses were based on surgical specimens and were used as the reference standard. Patients were categorized as having either pure DCIS or DCIS with an invasive component.

Tumor grade was recorded when available. Lymph node status was evaluated in patients who underwent SLNB.

### 2.5. Statistical Analysis

Statistical analyses were performed using IBM SPSS Statistics for Windows, Version 26.0 (IBM Corp., Armonk, NY, USA). Continuous variables are presented as mean ± standard deviation, and categorical variables as counts and percentages. The distribution of continuous variables was assessed using the Kolmogorov–Smirnov test.

Between-group comparisons were performed using the independent samples *t*-test for continuous variables and the Pearson chi-square test or Fisher’s exact test for categorical variables, as appropriate.

For the main analysis, imaging findings were grouped into three categories: no mass (absence on both modalities), mass detected on a single modality, and mass findings identified on both mammography and ultrasonography. Differences in invasive carcinoma rates across these groups were evaluated using the chi-square test.

All statistical tests were two-sided, and a *p*-value < 0.05 was considered statistically significant. Findings were interpreted primarily according to the magnitude and consistency of the observed associations rather than statistical significance alone. Cases with incomplete imaging or pathological data were excluded from the analysis, and missing values were not imputed. Because of the limited number of invasive events, the study was not designed to develop or validate a predictive model.

### 2.6. Ethics Approval

The study protocol was approved by the Institutional Ethics Committee (approval number: TABED 2-26-2214; approved on 15 April 2026) and conducted in accordance with the Declaration of Helsinki. Patient consent was waived due to the retrospective design of the study.

## 3. Results

A total of 125 patients were included, with a mean age of 52.6 ± 10.4 years. On final histopathology, 106 patients (84.8%) had pure ductal carcinoma in situ (DCIS), while 19 (15.2%) had an invasive component.

**Figure 2:**
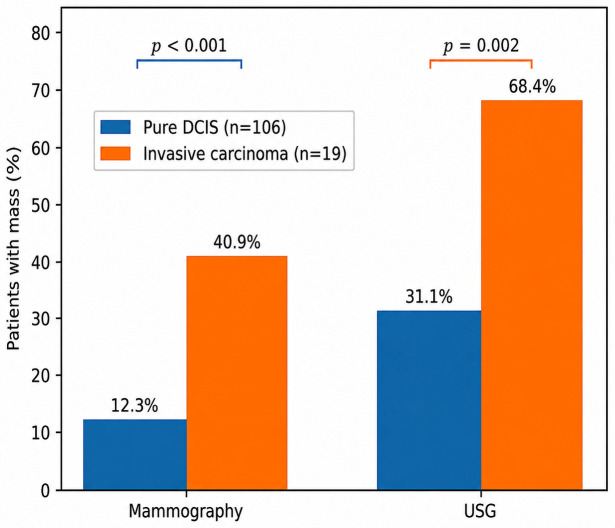
Frequency of mammographic mass findings and ultrasonographic solid masses according to invasive status. Percentages represent the proportion of patients with mammographic mass findings and ultrasonographic solid masses in the pure DCIS and invasive carcinoma groups.

Mass findings identified on both mammography and ultrasonography were more frequently observed in patients with invasive carcinoma (Table 1). On mammography, mammographic mass findings were present in 40.9% of patients with invasive carcinoma compared with 12.3% of those with pure DCIS (*p* < 0.001). On ultrasonography, solid masses were identified in 68.4% and 31.1% of patients, respectively (*p* = 0.002). These differences are illustrated in Figure 2.

Imaging findings were also categorized as no mass on either modality, mass detected on a single modality, or mass findings identified on both mammography and ultrasonography. Invasive carcinoma was observed in 6 of 71 patients (8.5%; 95% CI, 3.2–17.5%), 5 of 37 patients (13.5%; 95% CI, 4.5–28.8%), and 8 of 17 patients (47.1%; 95% CI, 23.0–72.2%) across these groups, respectively. Although the proportion of invasive carcinoma was highest in the latter group, the subgroup size was limited, and the findings should therefore be interpreted cautiously. Considerable overlap between imaging categories remained present.

Patients with invasive carcinoma were younger than those with pure DCIS (47.6 ± 10.1 vs. 53.4 ± 10.2 years, *p* = 0.027). Sentinel lymph node biopsy (SLNB) was performed in 44 patients (25 with pure DCIS and 19 with invasive carcinoma), and lymph node metastasis was detected in 13 (29.5%) (Table 2). Among patients who underwent SLNB, nodal involvement was more frequent in patients with invasive carcinoma than in those with pure DCIS (63.2% vs. 4.0%, *p* < 0.001).

Among patients with a preoperative diagnosis of DCIS, only one patient (0.9%) was found to have both invasive carcinoma and lymph node metastasis on final pathological examination. SLNB was performed selectively according to clinical and imaging findings, which may have influenced the observed rates of nodal involvement.

Other imaging findings, including microcalcifications, asymmetry, architectural distortion, complex masses, and ductal abnormalities, did not demonstrate a clear association with invasive carcinoma in this cohort (all *p* > 0.05). Mammographic breast density also did not differ between groups (*p* = 0.642).

Tumor grade was associated with both invasive carcinoma and lymph node metastasis (*p* < 0.001 for both). Detailed distributions of tumor grade according to invasion status and sentinel lymph node involvement are provided in Supplementary Table S1. Tumor grade information was available for 39 patients. Because tumor grade was not available for all patients and represented a postoperative pathological variable, it was not included in the primary imaging-based analysis and should therefore be interpreted descriptively.

Re-excision was more frequent in patients with invasive carcinoma than in those with pure DCIS (47.4% vs. 31.1%), although the difference was not statistically significant (*p* = 0.168).

## 4. Discussion

In this study, we examined the relationship between preoperative mammographic and ultrasonographic findings and the presence of invasive carcinoma in patients with ductal carcinoma in situ (DCIS).

The principal finding of the present study was that mass findings identified on both mammography and ultrasonography were more frequently observed in patients with invasive carcinoma. Previous studies have also reported a higher frequency of mass lesions in DCIS cases with invasive carcinoma [11,13]. However, these studies generally evaluated mammographic and ultrasonographic findings separately. In contrast, the present study assessed mass findings identified on both mammography and ultrasonography at the patient level rather than the lesion level.

Because of the limited number of invasive events and the resulting uncertainty of subgroup estimates, these findings should be interpreted cautiously. Without lesion-level matching, some mammographic and ultrasonographic findings may not have corresponded to the same lesion, particularly in multifocal or multicentric disease. Consequently, some degree of imaging misclassification cannot be excluded. Patients with invasive carcinoma were younger in our cohort; however, the clinical significance of this finding remains uncertain given the limited sample size.

Microcalcifications were not strongly associated with invasive carcinoma in this cohort. Previous studies have suggested that calcifications may more commonly reflect intraductal disease, whereas mass lesions may be observed more frequently in lesions with stromal invasion [11,13]. The absence of statistical significance should not necessarily be interpreted as evidence of no association.

Despite these associations, imaging findings alone do not appear sufficient to distinguish reliably between pure DCIS and DCIS with invasion. The substantial overlap between imaging categories suggests that imaging findings should be interpreted within the broader clinical context rather than as independent determinants of treatment decisions.

The retrospective design may have influenced imaging interpretation and clinical decision pathways. In addition, imaging evaluations were performed by a single experienced reader, and interobserver variability could not be assessed. Although interpretation by an experienced breast radiologist provided consistency throughout the study, assessment of mammographic masses, ultrasonographic solid masses, and BI-RADS findings remains subject to reader variability. Because imaging findings on both modalities were evaluated at the patient level rather than through formal lesion-by-lesion correlation, the apparent agreement between mammographic and ultrasonographic findings may have been overestimated in some cases, particularly in patients with multifocal or multicentric disease [11].

Because SLNB was performed selectively rather than universally, verification bias may have affected the observed nodal metastasis rates. Accordingly, the observed frequency of lymph node metastasis should not be considered representative of the entire study cohort. Occult invasive disease may also have contributed to nodal positivity in some patients initially classified as pure DCIS [5,6].

The single-center design and lack of external validation should also be considered when interpreting the findings. Furthermore, the simplified imaging classification used in this study did not include detailed assessment of lesion morphology, microcalcification morphology, or calcification distribution patterns. Therefore, the present study cannot determine whether specific microcalcification characteristics are associated with invasive carcinoma.

Larger prospective studies incorporating lesion-level imaging correlation may provide a clearer understanding of the significance of mass findings identified on both mammography and ultrasonography in DCIS.

## 5. Conclusions

Mass findings identified on both mammography and ultrasonography were more frequently observed in DCIS with invasive carcinoma; however, substantial overlap between imaging categories and the limited number of invasive events limit reliable patient-level discrimination. These findings should therefore be interpreted cautiously and confirmed in larger prospective studies incorporating lesion-level imaging correlation.

## Figures and Tables

**Table 1:** Radiological Findings Associated with Invasive Carcinoma.

Variable	Pure DCIS (*n* = 106)	DCIS with Invasive Carcinoma (*n* = 19)	*p*-Value
Age (mean + SD)	53.44 ± 10.19	47.58 ± 10.10	0.027
Microcalcifications, *n* (%)	79 (74.5%)	12 (63.2%)	0.305
Mammographic mass, *n* (%)	13 (12.3%)	9 (40.9%)	<0.001
Asymmetry, *n* (%)	38 (36.5%)	7 (36.8%)	0.980
Architectural distortion, *n* (%)	11 (10.4%)	2 (10.5%)	0.990
Ultrasonographic solid mass, *n* (%)	33 (31.1%)	13 (68.4%)	0.002
Ultrasonographic complex mass, *n* (%)	10 (9.4%)	4 (21.1%)	0.180
Ductal abnormality, *n* (%)	18 (17.0%)	3 (15.8%)	0.880

Abbreviations: SD, standard deviation.

**Table 2:** Sentinel Lymph Node Biopsy and Re-excision Outcomes.

Variable	DCIS (*n* = 106)	DCIS with Invasive Carcinoma (*n* = 19)	*p*-Value
SLN negative, *n* (%)	24 (96.0%)	7 (36.8%)	—
SLN positive, *n* (%)	1 (4.0%)	12 (63.2%)	<0.001
Re-excision yes, *n* (%)	33 (31.1%)	9 (47.4%)	0.168
Re-excision no, *n* (%)	73 (68.9%)	10 (52.6%)	—

## Data Availability

The data presented in this study are available from the corresponding author upon reasonable request.

## Supplementary Materials

The following supporting information can be downloaded at: https://www.mdpi.com/article/10.3390/diagnostics16132062/s1, Supplementary Table S1. Tumor grade distribution according to invasion status and sentinel lymph node involvement.

## Abbreviations

BI-RADS	Breast Imaging Reporting and Data System
DCIS	Ductal carcinoma in situ
SLNB	Sentinel lymph node biopsy
SPSS	Statistical Package for the Social Sciences
US	Ultrasonography
