# Supplementary material for: Patient-Level Mass Findings on Mammography and Ultrasonography in Ductal Carcinoma In Situ: Association with Invasive Carcinoma
Source: Diagnostics (Basel). 2026 Jul 1;16(13):2062. doi: 10.3390/diagnostics16132062 (PMC13360473; doi:10.3390/diagnostics16132062)
Supplement: Supplementary file 1 [file diagnostics-16-02062-s001.zip › diagnostics-4308515-supplementary.pdf]

Supplementary Table S1. Tumor grade distribution according to invasion status and sentinel lymph node involvement.

	Grade 1	Grade 2	Grade 3
SLN status			
SLN negative, n (%)	1 (3.8%)	3 (11.5%)	22 (84.6%)
SLN positive, n (%)	2 (16.7%)	7 (58.3%)	3 (25.0%)
Invasion status			
Invasive absent, n (%)	3 (13.0%)	0 (0.0%)	20 (87.0%)
Invasive present, n (%)	1 (6.3%)	10 (62.5%)	5 (31.3%)

Note: Tumor grade information was available only for a subset of patients and is presented descriptively.
